# Use of Pointwise Encoding Time Reduction with Radial Acquisition Magnetic Resonance Angiography for Coil Embolization

**DOI:** 10.2174/0115734056487521260819052008

**Published:** 2026-08-27

**Authors:** Jin Woo Kim, Hyunjung Kim, Jhii-Hyun Ahn, Jang Yoo, Sung Min Ko

**Affiliations:** 1 Department of Radiology, Wonju Severance Christian Hospital, Yonsei University Wonju College of Medicine, Wonju, South Korea;; 2 Department of Nuclear Medicine, Wonju Severance Christian Hospital, Yonsei University Wonju College of Medicine, Wonju, South Korea

**Keywords:** Digital subtraction angiography, PETRA MRA, TOF MRA, Intracranial aneurysm, Coil embolization, DSA, Ultrashort TE

## Abstract

**Introduction:**

It is imperative to perform imaging evaluation following endovascular coil treatment for cerebral aneurysms. Noninvasive vascular assessment using Magnetic Resonance Angiography (MRA), specifically Time-of-Flight (TOF) MRA, is commonly performed. Thus, this study assessed the diagnostic performance of Pointwise Encoding Time Reduction with Radial Acquisition (PETRA) MRA in patients who underwent simple coil embolization.

**Methods:**

Occlusion status, overall image quality, and treated-site image quality were compared across Digital Subtraction Angiography (DSA), TOF MRA, and PETRA MRA obtained during the same follow-up session after simple coil embolization of intracranial aneurysms.

**Results:**

Overall, 366 patients (median age: 61 ± 13 years) with 373 aneurysms underwent DSA, TOF MRA, and PETRA MRA following simple coil embolization. Occlusion status on PETRA MRA (85 cases, 91.4%) revealed stronger consistency with DSA than with TOF MRA (60 cases, 64.5%). Moreover, PETRA MRA demonstrated higher sensitivity for detecting residual neck and aneurysm (Raymond II and IIIa) compared with TOF MRA (Raymond II: 89% *vs*. 69%, *p* < 0.05; Raymond IIIa: 93% *vs*. 50%, *p* < 0.05). Notably, no significant differences were observed in overall or treated-site image quality between the techniques (*p = 0.9*).

**Discussion:**

PETRA MRA provides better visualization of residual flow within the coil mass and exhibits greater agreement with DSA than with TOF MRA. It also serves as an effective follow-up imaging modality, preserving image quality while enhancing diagnostic accuracy.

**Conclusion:**

PETRA MRA offers superior diagnostic performance compared with TOF MRA for follow-up evaluation following simple coil embolization of cerebral aneurysms.

## 1 INTRODUCTION

Endovascular treatment is the standard method for managing intracranial aneurysms, with coil embolization being its representative technique [1]. Although endovascular coil embolization prevents aneurysm rupture, coiled aneurysms recanalize in approximately 20% of cases, and some patients require follow-up for ≥ 10 years [2]. Furthermore, Digital Subtraction Angiography (DSA) with Three-Dimensional (3D) imaging provides high spatial resolution and remains the gold standard for evaluating or following treated aneurysms [3]. However, DSA is invasive and carries risks of thromboembolic complications, radiation exposure, and reactions to iodine-based contrast agents [4]. Thus, noninvasive vascular screening using Magnetic Resonance Angiography (MRA) is widely performed. Notably, previous studies have demonstrated the usefulness of Time-of-Flight (TOF) MRA for detecting aneurysm recurrence [5-7].

Recurrence or residual flow assessment in coiled aneurysm and visualization of in-stent flow after coil embolization are limited by radiofrequency shielding and susceptibility artifacts [8, 9]. Ultrashort Echo Time (UTE) imaging helps overcome these artifacts caused by coil and stent placement [10]. Previous studies have demonstrated that UTE-based MRA improves visualization of post-endovascular treatment sites compared with TOF MRA [12-16]. Although Pointwise Encoding Time Reduction with Radial Acquisition (PETRA) provides a higher signal-to-noise ratio than conventional UTE techniques due to shorter k-space encoding and arterial spine labeling [17, 18], most evidence focuses on stent-assisted coil embolization. Artifacts from the coil bundle can hinder accurate evaluation of blood flow within the coil in cases of recurrence following simple coil embolization, which is the most commonly performed endovascular treatment, specifically for Raymond III recurrences. To date, only a few studies have investigated the use of PETRA MRA for simple coil embolization, and direct comparisons with DSA are scarce or involve relatively small patient cohorts [11-14]. This study evaluated the diagnostic performance of PETRA MRA versus TOF MRA for assessing aneurysm occlusion status after simple coil embolization with DSA as the reference standard.

## 2 MATERIALS AND METHODS

### 2.1 Patients

We reviewed data from consecutive patients who underwent PETRA MRA and TOF MRA in the same session after simple coil embolization for intracranial aneurysms from January 2020 to December 2024. Patients were enrolled in the study only if the interval between DSA and MRA (TOF and PETRA) imaging was < 3 months. Exclusion criteria were inability to obtain TOF and PETRA images simultaneously, an interval between MRA and DSA imaging > 3 months, or unsuitable images due to other causes, *e.g*., clip- or stent-assisted coil embolization for other aneurysms, Onyx treatment for arteriovenous malformations or dural arteriovenous fistulas, and deep brain stimulation. To ensure a sufficient sample with representative participants, the sample size was determined using strict inclusion criteria based on the number of imaging tests and procedures performed, as well as prior neuroimaging studies demonstrating PETRA imaging utility (Fig. **1**). This retrospective study was approved by the Institutional Review Board of Yonsei University Wonju Christian Hospital, South Korea (Approval No.: CR324137, Approval Date: March 2025), and written consent was waived in compliance with the revised Declaration of Helsinki and regulations regarding retrospective data.

### 2.2 Imaging Acquisition

PETRA MRA and TOF MRA images were acquired using a 3-Tesla Magnetic Resonance Imaging (3T MRI) scanner (Vida, Siemens, Erlangen, Germany) with a 64-channel head and neck coil. TOF MRA scan parameters were as follows: Repetition Time/Echo Time (TR/TE), 21/3.69 ms; flip angle, 15°; Field of View (FOV), 144 × 230; matrix, 520 × 300; voxel size, 0.5 × 0.5 × 0.5 mm^3^; section thickness, 0.5 mm; Number of Excitations (NEX), 1; bandwidth, 186 Hz/pixel; acquisition time, 6 min 10 s. The PETRA MRA scan parameters were as follows: TR/TE, 3.32/0.07 ms; flip angle, 6°; FOV, 200 × 200; matrix, 340 × 340; voxel size, 0.72 × 0.72 × 0.72 mm^3^; section thickness, 0.72 mm; NEX, 1; bandwidth, 403 Hz/pixel; acquisition time, 6 min 21 s. PETRA MRA images were generated from two acquisitions, with and without a slice-selective saturation pulse for the carotid artery. Moreover, the maximum-intensity projections for PETRA and TOF were reconstructed in the same manner as DSA images.

DSA images were obtained using the biplane angiographic system (Allura Clarity, Philips, Massachusetts, USA) with contrast media injection. Intracranial arteries were displayed in at least two projections (anteroposterior and lateral views) and with 3D rotational angiography. DSA parameters were as follows: matrix, 512 × 512; FOV, 110 × 110 mm; temporal resolution, 5 frames/s.

### 2.3 Image Analysis

All data were anonymized *via* random number assignments, and all images were independently evaluated randomly by two experienced neuroradiologists (7 and 13 years of experience, respectively). Notably, TOF, PETRA, and DSA imaging were evaluated by each reader in a blinded manner at 1-week intervals to assess recurrence and image quality. Residual flow and aneurysm occlusion were assessed as follows, according to the Modified Raymond-Roy Occlusion Classification: I, complete obliteration; II, residual neck; IIIa, residual aneurysm opacification within the coil interstices; IIIb, residual aneurysm opacification between the coil mass and aneurysm wall [19]. Furthermore, recurrence on TOF and PETRA images was evaluated using Raymond-Roy classification, with DSA as the reference standard. Disagreement between the two neuroradiologists regarding the residual flow and aneurysm occlusion was resolved *via* consensus. Overall image quality was subjectively rated using a 4-point Likert scale based on diagnostic confidence and image blurring, as follows: 1) unacceptable (nondiagnostic); 2) acceptable (diagnostic with minor limitations); 3) good (diagnostic without significant limitations); and 4) excellent (optimal diagnostic quality) [20]. Visualization of the treatment site was similarly scored for the clarity of blood-flow signals within the coil.

### 2.4 Statistical Analysis

Categorical variables are presented as counts with frequencies (%), and continuous variables are presented as means ± standard deviations or medians with interquartile ranges. The Wilcoxon signed-rank test and Bowker’s test were used to compare scores between TOF MRA and PETRA MRA, and the Mann-Whitney U test was applied for subgroup comparisons. Furthermore, interobserver agreement was assessed using weighted kappa values for image quality and treated-site visualization. All statistical analyses were performed using SPSS (v26.0; IBM, Armonk, NY) and MedCalc for Windows (v18.11.3; MedCalc Software, Mariakerke, Belgium); *P*-values of <0.05 were considered statistically significant.

## 3 RESULTS

Overall, 366 patients (median age, 61 ± 13 years) with 373 aneurysms (2 patients had 2 aneurysms, and 1 patient had 3 aneurysms) who underwent DSA, TOF MRA, and PETRA MRA after simple coil embolization between January 2020 and December 2024 were retrospectively enrolled in this study. Based on DSA of the 373 aneurysms, 280 (75.1%) were classified as Raymond I, 66 (17.7%) as Raymond II, 18 (4.8%) as Raymond IIIa, and 9 (2.4%) as Raymond IIIb (Table **1**). In TOF MRA, residual flow was observed in 43 (11.5%) cases with Raymond II, 9 (2.4%) with Raymond IIIa, and 8 (2.1%) with Raymond IIIb; in PETRA MRA, it occurred in 59 (15.8%) cases with Raymond II, 17 (4.6%) with Raymond IIIa, and 9 (2.4%) with Raymond IIIb (Table **2**). Aneurysm occlusion status on PETRA MRA (85 cases, 91.4%) was more consistent with DSA than with TOF MRA (60 cases, 64.5%) (Fig. **2**). Notably, PETRA MRA demonstrated higher sensitivity for detecting residual flow (Raymond II and IIIa) than TOF MRA (Raymond II: 89% *vs*. 69%, *p* < 0.05; Raymond IIIa: 93% *vs*. 50%, *p* < 0.05).

PETRA MRA exhibited the highest sensitivity for detecting residual neck (Raymond II: PETRA MRA *vs*. TOF MRA, 0.89 *vs*. 0.65, *p* = 0.033) and for diagnosing residual aneurysm (Raymond IIIa: 0.93 *vs*. 0.50, *p* = 0.001). Notably, PETRA MRA performed exceptionally well in identifying residual aneurysm opacification within the coil interstices. The diagnostic performance of PETRA MRA and TOF MRA is summarized in Table **3**.

Regarding overall image quality, PETRA MRA scores were slightly lower than TOF MRA scores (observer 1: 3.2 ± 0.5 *vs*. 3.4 ± 0.6; observer 2: 3.4 ± 0.4 *vs*. 3.5 ± 0.5); however, the differences were not statistically significant. In PETRA MRA, which requires a subtraction process, low image scores of 1-2 were relatively frequently distributed, and most of these scores were artifacts caused by motion. Interobserver agreement was moderate for PETRA MRA (weighted kappa, 0.72 [0.49-0.95]) and TOF MRA (0.77 (0.53-1.00). Treatment-site image quality was scored similarly by the observers (observer 1: 3.4 ± 0.5 *vs*. 3.5 ± 0.3; observer 2: 3.5 ± 0.3 *vs*. 3.5 ± 0.4), with moderate interobserver agreement for PETRA MRA (0.79 (0.51-1.00) and TOF MRA (0.84 (0.55-1.00) (Table **4**).

## 4 DISCUSSION

Herein, PETRA MRA demonstrated better visibility of treated aneurysms and higher sensitivity for detecting residual flow, with no substantial differences in overall image quality compared to TOF MRA. Moreover, detection of residual neck and aneurysm *via* PETRA MRA was better than that *via* TOF MRA, using DSA as the reference standard (PETRA *vs*. TOF (Raymond II: 89% *vs*. 69%, *p* < 0.05; Raymond IIIa: 93% *vs*. 50%, *p* < 0.05)). Previous studies have also reported PETRA’s effectiveness in diagnosing aneurysm recurrence and evaluating image quality [12, 14, 21]. However, these studies were conducted with relatively small samples and did not directly compare PETRA and DSA. Our study further confirmed the diagnostic value of PETRA through numerous direct comparisons with DSA, the gold standard for evaluating cerebral aneurysms.

Previous studies on UTE MRA in endovascularly treated aneurysms have largely focused on stent-assisted coil embolization [12, 21]. In stent-assisted procedures, assessing recurrence or residual flow and visualizing in-stent flow is limited by radiofrequency shielding and susceptibility artifacts [9, 22]. Even in simple coil embolization, the coil mass can generate magnetic susceptibility or blooming artifacts, obstructing visualization and interpretation of residual flow [15, 16]. These artifacts arise from differences in magnetic susceptibility among the stent strut, coil, and surrounding tissue. Considering UTE’s low dephasing time, susceptibility artifacts are often minimal or absent [12, 13, 15].

PETRA MRA can be considered a variant of UTE imaging. However, unlike conventional UTE techniques, it provides more complete filling of the central k-space, thereby minimizing data loss and providing more stable, higher-quality images [15-18]. The present study focused on the use of PETRA imaging for follow-up after simple coil embolization, which is the most commonly performed endovascular treatment. PETRA MRA demonstrated robust diagnostic capabilities, specifically for residual aneurysms. PETRA MRA is known to resist flow dephasing through its combination of radial and Cartesian acquisition and UTE, potentially reducing internal flow disturbance within the aneurysmal cavity and improving visualization [23-25]. These advantages allow PETRA to more accurately visualize residual or recurrent flows within the coil mass without obscuration or distortion.

A previous study comparing TOF and PETRA in intracranial aneurysms reported that PETRA improved the homogeneity of intra-aneurysmal signal intensity [25]. Furthermore, UTE MRA may blur vessel edges because of radial sampling from the vessel center outward [20]. Conversely, PETRA MRA reduces flow-related phase contrast artifacts by combining radial and orthogonal acquisition techniques [15, 16]. PETRA MRA shows a sharper boundary between aneurysms and background tissue than TOF MRA [25]. Thus, improved aneurysm visualization appears mainly due to suppression of background tissue and venous flow in subtraction-based PETRA MRA. TOF MRA uses a flow-compensated gradient-echo sequence that generates images by radiofrequency excitation of unsaturated protons flowing into a saturated stationary imaging plane [11]. Consequently, vessels running parallel to the scan plane may be difficult to visualize because of signal saturation. Additionally, signal loss due to intraluminal vortices within an aneurysm can compromise accurate depiction of complex turbulent flow, particularly in cases of residual or recurrent aneurysms [12, 14]. Conversely, PETRA MRA is largely insensitive to saturation effects and independent of vessel orientation, thereby reducing the eddy-current effects within the aneurysm and reliably depicting minimal residual or recurrent blood flow [26, 27]. PETRA’s inside-out radial acquisition reduces phase dispersion of labeled blood flow signals in the voxel space, decreasing PETRA MRA sensitivity to turbulent flow [27, 28]. Prior studies suggested that PETRA more accurately depicts cerebrovascular diseases, such as stenotic arterial occlusions, large cerebral aneurysms, and Moyamoya disease, by reducing acoustic noise, enhancing signal uniformity, and improving venous signal suppression [26-28]. In the present study, PETRA provided clearer images and closer resemblance to DSA in cases with residual flow (Fig. **3**), accurately displaying flow within the coil structure.

### 4.1 Limitations

This study had several limitations, the first being a retrospective design and its performance at a single institution using a single 3T MRI scanner, which introduced potential selection bias. Raymond type III (IIIa + IIIb, n = 27), considered a major recurrence type, was observed in relatively few cases. Regarding aneurysm location, the majority (61.4%) were found in the distal internal carotid artery, indicating limited variability. To our knowledge, we evaluated the highest number of intracranial PETRA images in a single study to date; nevertheless, multicenter research is required. Second, PETRA MRA was not compared with contrast-enhanced MRA. However, contrast-enhanced MRA carries risks associated with contrast injection, and previous studies have suggested that TOF MRA provides sufficient diagnostic performance for simple coil embolization follow-up [5]. Third, PETRA imaging based on subtraction requires relatively long scan times, with motion and subtraction artifacts remaining possible. Future research should aim to optimize imaging parameters and reduce scan times.

## CONCLUSION

In conclusion, this study demonstrated the superior diagnostic performance of PETRA MRA over TOF MRA in detecting residual neck or aneurysm after simple coil embolization for cerebral aneurysms. Therefore, PETRA MRA may be used as an alternative imaging modality for follow-up of treated aneurysms. Nonetheless, for broader clinical application, larger multicenter studies with more cases of major recurrences are required.

## Figures and Tables

**Fig. (1):**
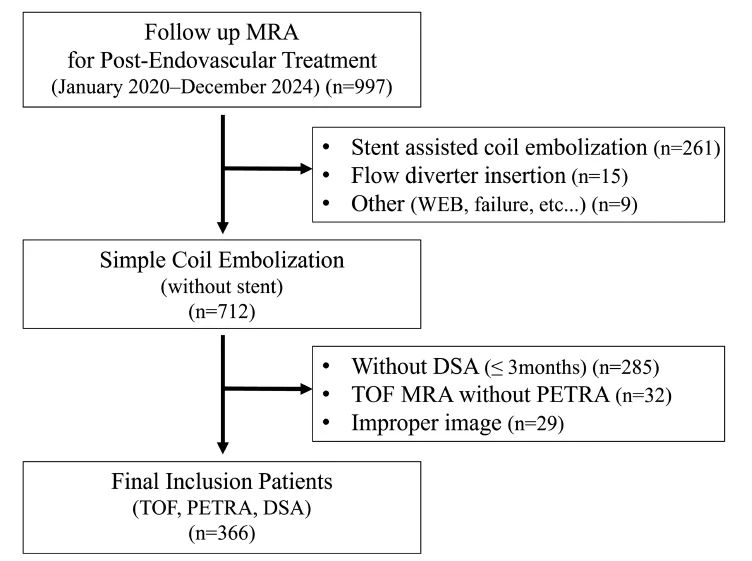
Study design flowchart. **Abbreviations:** MRA: Magnetic Resonance Angiography; PETRA: Pointwise Encoding Time Reduction with Radial Acquisition; TOF: Time-of-Flight; DSA: Digital Subtraction Angiography

**Fig. (2):**
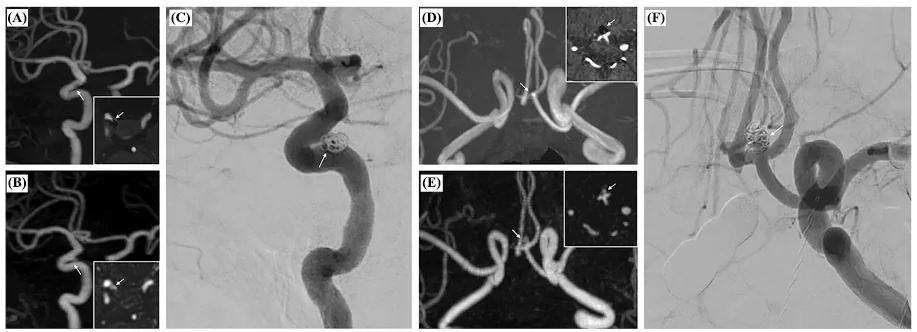
Images of two patients with treated intracranial aneurysms. (**A**-**C**) A 65-year-old woman with a treated aneurysm of the right paraclinoid internal carotid artery. Time-of-Flight Magnetic Resonance Angiography (TOF MRA) did not detect the residual neck (arrow) of the aneurysm (A, source image, dashed arrow), and pointwise encoding time reduction with radial acquisition magnetic resonance angiography (PETRA MRA) (**B**) and Digital Subtraction Angiography (DSA) (**C**) clearly demonstrated the residual neck (arrow). (**D**-**F**) A 57-year-old woman with a treated aneurysm of the anterior communicating artery. TOF MRA did not detect the residual aneurysm (D, source image, dashed arrow), whereas PETRA MRA (**E**) and DSA (**F**) clearly showed the residual aneurysm (arrow).

**Fig. (3):**
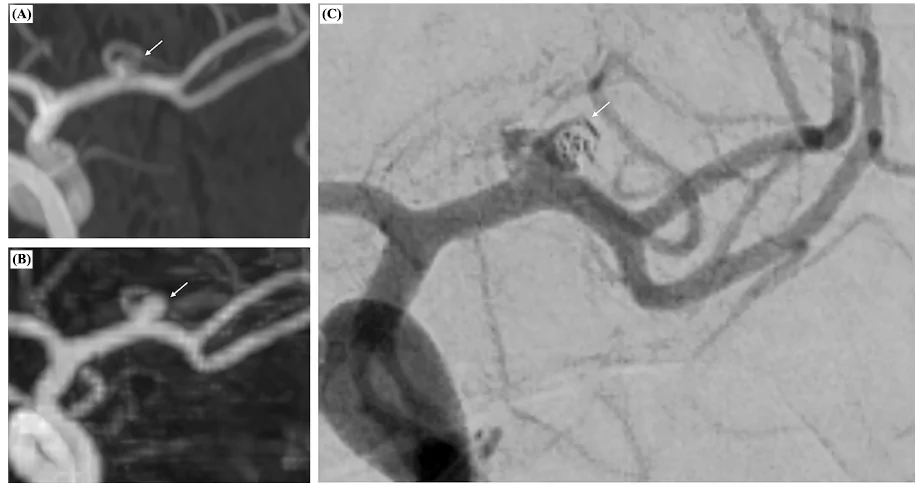
Images from a 62-year-old man with a left middle cerebral artery aneurysm who underwent coil embolization. Recurrence was suspected on follow-up Magnetic Resonance Angiography (MRA), and Digital Subtraction Angiography (DSA) was performed for confirmation. Time-of-Flight Magnetic Resonance Angiography (TOF MRA) (**A**) and Pointwise Encoding Time Reduction with Radial Acquisition Magnetic Resonance Angiography (PETRA MRA) (**B**) detected the recurrent aneurysm. Compared with DSA (**C**), PETRA MRA provided clearer visualization, with findings more closely resembling those of DSA.

**Table 1:** Baseline patient characteristics.

**Characteristics**		**Simple Coil Embolization**
No. of Patients		366
No. of Aneurysms		373
Age: years		61 ± 13
Sex: female		211 (58%)
Location: count (percentage)	-	-
	Distal ICA	229 (61.4%)
	ACA	64 (17.2%)
	MCA	43 (11.5%)
	BA	21 (5.5%)
	Distal VA	9 (2.5%)
	PCA	4 (1.1%)
	PICA	2 (0.5%)
	SCA	1 (0.3%)
Aneurysm	Height	5.1 ± 3.8
Duration of follow-up MRA to DSA (days)		71 ± 3.3
Modified Raymond-Roy Occlusion Classification, count (percentage)	I	280 (75.1%)
	II	66 (17.7%)
	IIIa	18 (4.8%)
	IIIb	9 (2.4%)

**Abbreviations:** ICA: Internal Carotid Artery; ACA: Anterior Cerebral Artery; MCA: Middle Cerebral Artery; BA: Basilar Artery; VA: Vertebral Artery; PCA: Posterior Cerebral Artery; PICA: Posterior Inferior Cerebellar Artery; SCA: Superior Cerebellar Artery.

**Table 2:** Aneurysm occlusion status on PETRA MRA and TOF MRA.

**DSA**	**PETRA**				**TOF**			
	**Raymond I (n = 288)**	**Raymond II (n = 59)**	**Raymond IIIa (n = 17)**	**Raymond IIIb (n = 9)**	**Raymond I (n = 313)**	**Raymond II (n = 43)**	**Raymond IIIa (n = 9)**	**Raymond IIIb (n = 8)**
Raymond I (n = 280)	276	1	0	3	279	0	0	1
Raymond II (n = 66)	9	57	0	0	23	42	0	1
Raymond IIIa (n = 18)	1	0	17	0	7	1	9	1
Raymond IIIb (n = 9)	2	1	0	6	4	0	0	5

**Table 3:** Diagnostic performance of TOF MRA and PETRA MRA for assessing aneurysmal occlusion status.

**DSA**	**PETRA**					**TOF**				
	**Sensitivity**	**Specificity**	**PPV**	**NPV**	**Accuracy**	**Sensitivity**	**Specificity**	**PPV**	**NPV**	**Accuracy**
Raymond I	0.99	0.88	0.96	0.95	0.92	0.99	0.63	0.89	0.98	0.89
Raymond II	0.89	0.99	0.97	0.97	0.98	0.69	0.99	0.97	0.93	0.94
Raymond IIIa	0.93	1.0	1.0	0.99	0.99	0.50	1.0	1.0	0.97	0.97
Raymond IIIb	0.66	0.99	0.66	0.99	0.97	0.55	0.99	0.62	0.98	0.98

**Abbreviations:** PPV: Positive Predictive Value; NPV: Negative Predictive Value.

**Table 4:** Image quality comparison between PETRA MRA and TOF MRA.

-	Observer	**PETRA (n = 373)**				**TOF (n = 373)**				*P-*value
		**1**	**2**	**3**	**4**	**1**	**2**	**3**	**4**	-
Overall Image Quality	1	8	40	184	141	1	24	161	187	0.91
	2	5	29	167	172	1	18	162	192	0.96
	Agreement	0.72 (0.49-0.95)				0.77 (0.53-1.00)				
Treated-site Image Quality	1	7	30	173	163	1	19	157	196	0.95
	2	5	28	160	180	1	20	164	188	0.99
	Agreement	0.79 (0.51-1.00)				0.84 (0.55-1.00)				

## Data Availability

Additional data used in the study may be acquired from the corresponding author [S.M.K] upon reasonable request.

## LIST OF ABBREVIATIONS

MRA: Magnetic Resonance Angiography
TOF: Time-of-Flight
PETRA: Pointwise Encoding Time Reduction with Radial Acquisition
DSA: Digital Subtraction Angiography
UTE: Ultrashort Echo Time

## STANDARDS OF REPORTING

STROBE guidelines were followed.
